# Doxorubicin–transferrin conjugate alters mitochondrial homeostasis and energy metabolism in human breast cancer cells

**DOI:** 10.1038/s41598-021-84146-4

**Published:** 2021-02-25

**Authors:** Paulina Wigner, Krzysztof Zielinski, Magdalena Labieniec-Watala, Agnieszka Marczak, Marzena Szwed

**Affiliations:** Department of Medical Biophysics, Institute of Biophysics, Faculty of Biology and Environmental Protection, University of Lodz, Lodz, Poland

**Keywords:** Biochemistry, Biophysics, Biotechnology, Cancer, Cell biology, Chemical biology, Drug discovery, Molecular biology

## Abstract

Doxorubicin (DOX) is considered one of the most powerful chemotherapeutic agents but its clinical use has several limitations, including cardiomyopathy and cellular resistance to the drug. By using transferrin (Tf) as a drug carrier, however, the adverse effects of doxorubicin as well as drug resistance can be reduced. The main objective of this study was to determine the exact nature and extent to which mitochondrial function is influenced by DOX–Tf conjugate treatment, specifically in human breast adenocarcinoma cells. We assessed the potential of DOX–Tf conjugate as a drug delivery system, monitoring its cytotoxicity using the MTT assay and ATP measurements. Moreover, we measured the alterations of mitochondrial function and oxidative stress markers. The effect of DOX–Tf was the most pronounced in MDA-MB-231, triple-negative breast cancer cells, whereas non-cancer endothelial HUVEC-ST cells were more resistant to DOX–Tf conjugate than to free DOX treatment. A different sensitivity of two investigate breast cancer cell lines corresponded to the functionality of their cellular antioxidant systems and expression of estrogen receptors. Our data also revealed that conjugate treatment mediated free radical generation and altered the mitochondrial bioenergetics in breast cancer cells.

## Introduction

Iron ions play an essential role during breast cancer initiation and tumor progression^1,2^. First, as a key player in the Fenton reaction, iron augments the production of oxygen free radicals and consequently triggers carcinogenic mutations. Second, as a crucial growth factor, iron is a cofactor for ribonucleotide reductase—an enzyme involved in DNA synthesis of rapidly dividing cells^3^. Iron uptake by normal and malignant mammary epithelial cells is performed mostly via transferrin (Tf)-dependent endocytosis^4^. In this pathway, transferrin with two iron ions forms a complex with Tf receptor TfR1, CD71) and is engulfed by the cells as an endosome. Overexpression of endogenous TfR has been described in many different cancer types including those of lymph nodes^5,6^, lung, pancreas, or colon^6^. An immunohistochemical study performed by Wrba et al.^7^ confirmed that 88% of primary breast carcinomas were strongly positive for TfR and the percentage of cells displaying immunoreactivity was significantly correlated with the grade of tumor. Moreover, experiments carried out on MCF-7 cell line—an in vitro model of endocrine responsive, estrogen receptor (ER) + breast cancer—revealed a possible association between TfR and ER signaling. In accordance with this, high TfR1 expression was connected with poor response to tamoxifen treatment among women with triple-negative breast cancer^8^.

Keeping in mind which cellular compartments are in strong need of iron ions, it has recently emerged that the inhibition of mitochondrial bioenergetics may be a new, anticancer tool for breast tumor chemotherapy. This idea is supported by Warburg’s observation of the growth in ATP production and limited oxidative phosphorylation (OXPHOS) in human tumor cells^9^. Even though, the Warburg effect has been widely recognized, some studies have demonstrated that mitochondria in cancer cells are able to perform OXPHOS along with glycolysis^10^. With the reference to the breast cancer cells displaying a high demand for energy, it seems likely that compounds directed at mitochondrial bioenergetics can sensitize these cells to induction of programmed cell death. Therefore, there is a strong need to investigate antimitochondrial properties of the present and future anticancer drugs.

Doxorubicin (DOX) is one of the key chemotherapeutic agents for early and advanced breast cancer, along with other approaches such as surgery, radiotherapy and immunotherapy^11^. The major molecular mechanism responsible for the anticancer activity of DOX includes DNA lesions^12^, induction of apoptosis by activation of p53 protein and failure of mitochondrial function. It has been shown that the cellular DOX accumulation increases production of reactive oxygen species (ROS) and triggers damage to mitochondrial respiration related to protein synthesis and lipid peroxidation, release of cytochrome c from mitochondria, and activation of caspases^13^. Nevertheless, under conditions of continuous oxidative stress, DOX treatment can also affect normal noncancerous tissues and thereby impair healthy organs such as heart, liver or kidney. This limitation reduces the effective dose of DOX applied in breast cancer treatment but also deteriorates the quality of life of cancer patients^14^.

Here, we propose that a novel approach to breast cancer treatment would be the use of TfR as a target to deliver DOX directly into cancer cells. Keeping in mind that TfR expression is up to five times higher in malignant compared to normal tissue^10^, DOX–Tf conjugate should allow us to increase the intracellular concentration of drugs in breast cancer cells and thus help to overcome their chemoresistance^15^. We previously demonstrated that conjugation of DOX with Tf substantially increased cytotoxicity in both DOX-sensitive and DOX-resistant human leukemia cells, as well as those derived from solid tumor cells^16^. Our recent study indicated as well that Tf-bound DOX triggers programmed cell death with the engagement of the TRAIL-dependent, extrinsic pathway of apoptosis. The involvement of TNF-α and other cytokines suggests that pro-inflammatory effects of the conjugate are closely related with its cytotoxicity^17^.

However, little is known about DOX–Tf conjugate toxicity towards human breast cancer cells, especially with the reference to those cells that express ERs variously. We hypothesized that this novel DOX delivery system would be more effective for intracellular accumulation, mitochondrial targeting and cytotoxicity of DOX in cancer cells. To the best of our knowledge, no research has been reported so far concerning the application of DOX–Tf conjugate against breast cancer cell mitochondria. Thus, in this study we examined the efficacy of DOX–Tf conjugate against two breast cancer cell lines. In parallel, the experiments were performed on HUVEC-ST, an in vitro model of human endothelial cells. The breast cell lines differ in their characteristics and molecular profile in the following way: MCF-7 (luminal A^+^, ER^+^, PR^+^, Her2^+^) and MDA-MB-231 (claudin low, ER − , PR − , Her2 −)^18^. Regarding the expression of (TfR)1 within these two cell lines, MDA-MB-231 has a much larger number of receptors (24 ± 3 nmol/L) than MCF-7 (6.1 ± 0.2 nmol/L)^19^. Moreover, previously published clinical data support the statement that triple negative breast cancer tissue showed significantly higher CD71 protein expression in comparison to different types of tumor^20^.

**Figure 1:**
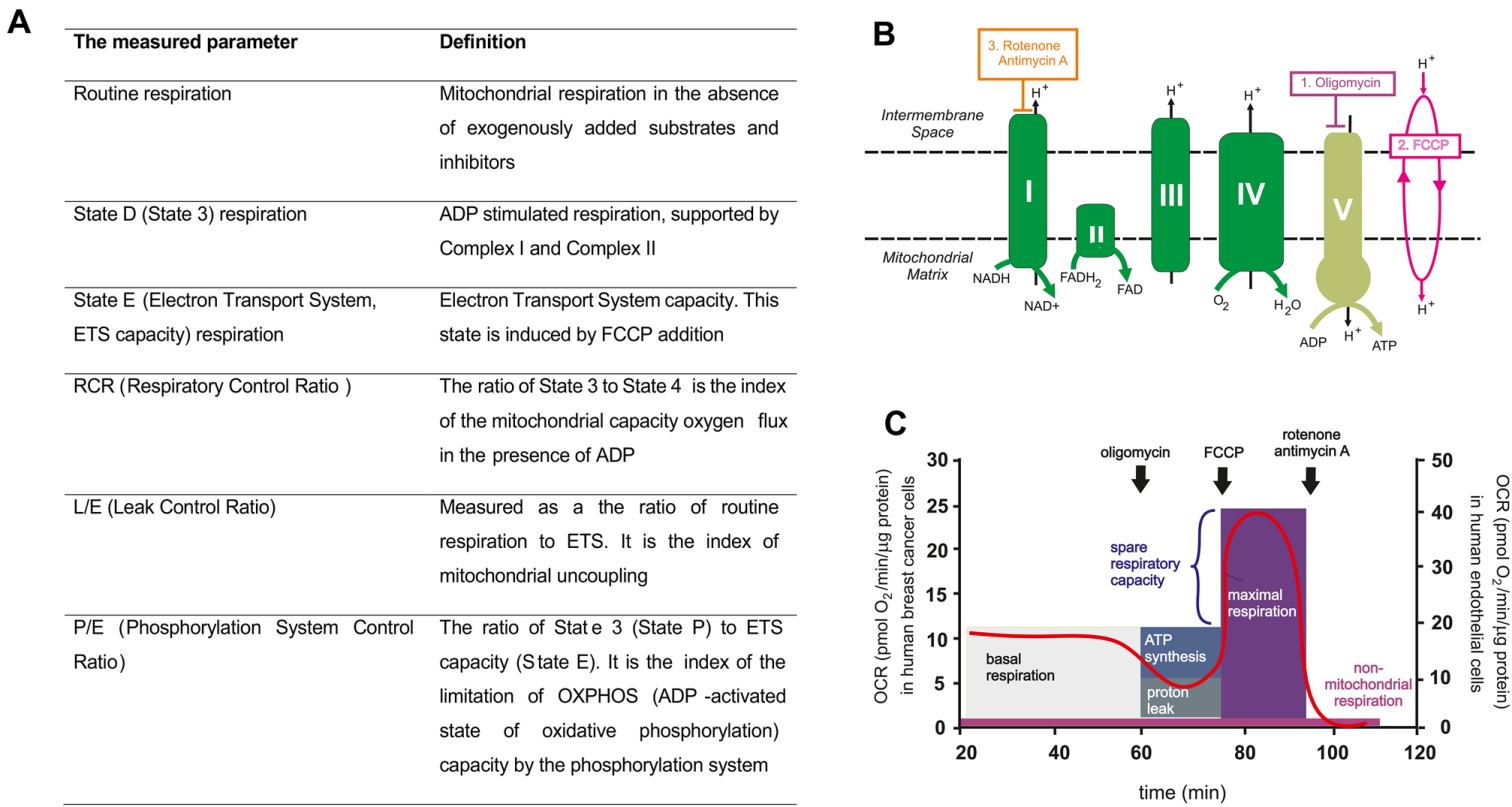
Principle of the mitochondrial stress assay. (**A**) Proposed definitions for cellular bioenergetics based on oxygen consumption rate (OCR). (**B**) Targets of each compound in the electron transport chain. (**C**) OCR levels refer to breast cancer and normal cells (left and right y axis, respectively) versus time (x axis). Injection of the three compounds oligomycin, FCCP, and antimycin A/rotenone delimited four time intervals within each of which OCR was roughly constant. Oligomycin is a specific inhibitor of the ATPase complex V and prevents protons from crossing the membrane through this complex to phosphorylate ADP. Oligomycin was used here to inhibit cellular respiration completely. FCCP is a mitochondrial uncoupler that allows protons to leak freely across the mitochondrial membrane and uncouples oxygen consumption from phosphorylation of ADP. Antimycin A is a specific inhibitor of Complex III, and rotenone is a specific inhibitor of Complex I. They were used together to block all mitochondrial respiration even in the presence of an uncoupler such as FCCP.

Here, our study paid particular attention to the influence of DOX–Tf conjugate on the basic parameters of mitochondrial respiration. The data indicate that DOX–Tf conjugate is a promising drug delivery system and further efficacy studies on experimental and preclinical models of breast cancer should be performed (Fig. 1).

## Results

### DOX–Tf induces high toxicity in human breast cancer cells

**Figure 2:**
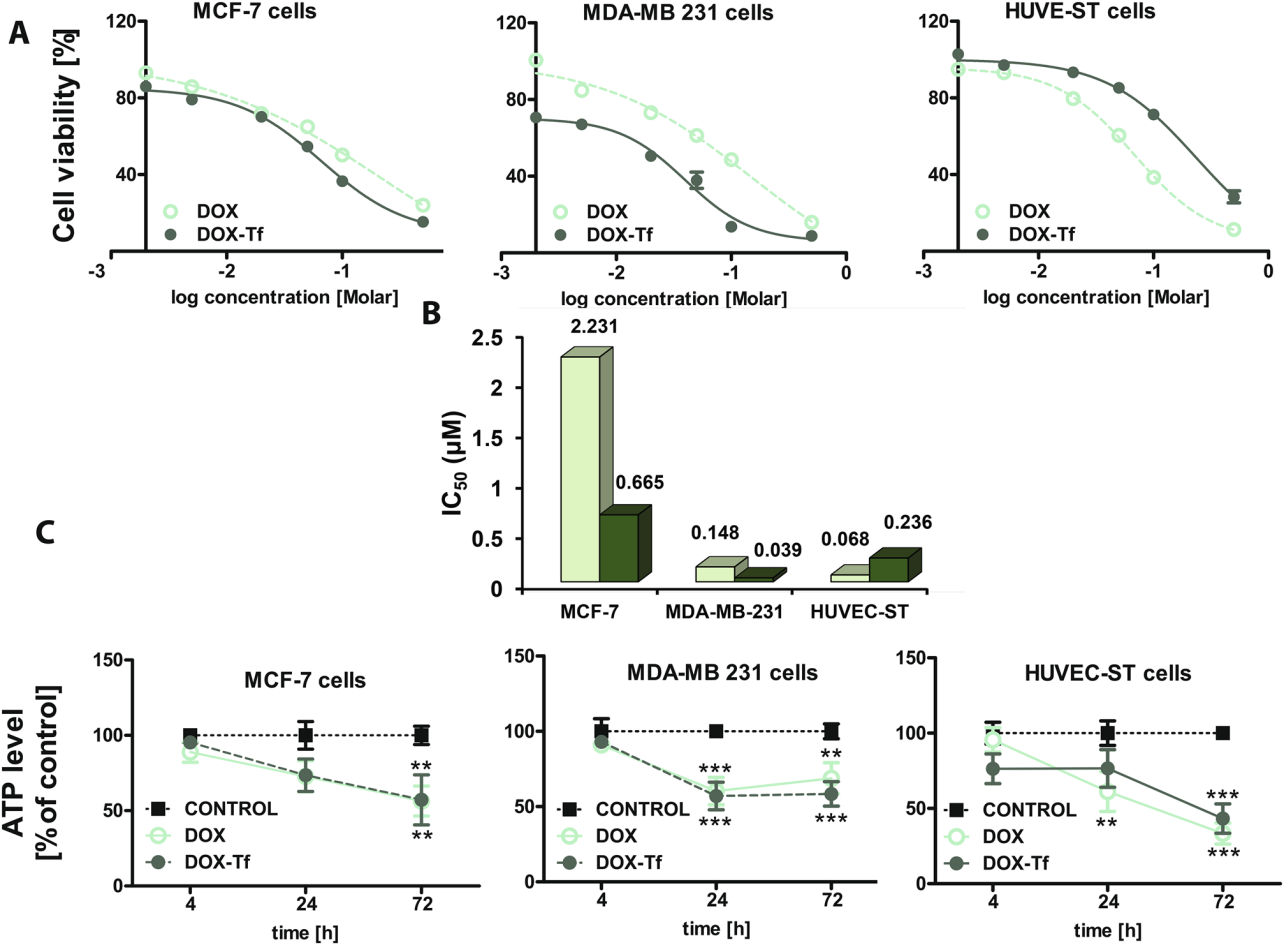
Comparisons of cytotoxicity of free DOX and DOX–Tf conjugate in breast cancer and endothelial cell lines. (**A**) Viability of MCF-7, MDA-MB-231 and HUVEC-ST cells measured by MTT assay after incubation of the cells for 72 h with increasing concentrations of DOX and DOX–Tf. The values are mean ± SD of five independent experiments with six replicates in each experiment. (**B**) IC_50_ values (calculated on basis of cell viability curves) for free DOX and DOX–Tf conjugate in MCF-7, MDA-MB 231 and HUVEC-ST cells. (**C**) Cellular ATP level in MCF-7, MDA-MB-231, and HUVEC-ST cells treated with IC_50_ concentrations of DOX alone or DOX–Tf for 4, 24 and 72 h. All values were normalized to untreated, control cells, taken as 100%. Data are expressed as mean ± SD (n = 6). Asterisks refer to the level of significance @@(**p < 0.01, ***p < 0.001).

DOX–Tf and DOX cytotoxicity was compared in two breast cancer cell lines and noncancer HUVEC-ST cells. There was a striking difference between the toxicity of these two forms of DOX. Cell viability was evaluated 72 h after treatment with DOX–Tf and DOX using the MTT assay. The cell lines exhibited a significantly different cytotoxic response to free DOX and DOX–Tf conjugate (Fig. 2A), which was confirmed by calculation of IC_50_ values (Fig. 2B). The two breast cancer cell lines were consistently more sensitive to DOX–Tf than to DOX, whereas normal endothelium cells were, significantly, 3.5-fold more resistant to DOX–Tf conjugate than to DOX alone. A striking difference in DOX–Tf conjugate cytotoxicity was observed between estrogen-positive and -negative breast cancer cell lines. MCF-7 cells seemed to be less sensitive to free DOX and DOX–Tf conjugate than MDA-MB-231 cells were. DOX–Tf triggered a 17-fold decrease in the metabolic activity of MDA-MB-231 cells measured by MTT reduction to formazan in comparison to MCF-7 cells. DOX–Tf conjugate was less cytotoxic against normal endothelial cells than against each of the breast cancer cell lines; even the triple-negative breast cancer cell line MDA-MB-231.

To analyze whether cytotoxicity of DOX–Tf was related to inhibition of cellular metabolism, we measured intracellular ATP level. The two breast cancer cell lines and HUVEC-ST cells were treated with DOX–Tf conjugate or free DOX (both at IC_50_ concentration) and then incubated for 4, 24 and 72 h. Even though there was no large difference between estrogen-positive and -negative breast cancer cells, ATP level was gradually diminished in MCF-7 and HUVEC-ST cells treated with free DOX and DOX–Tf in a time-dependent manner (Fig. 2C). The highest changes in luminescence intensity were noted after 72 h incubation with DOX–Tf (about 50% for MCF-7 and MDA-MB-231 cells). Human endothelial cells were more sensitive to DOX treatment than the breast cancer cells were, which was in agreement with the MTT assay. In addition, DOX–Tf appeared to decrease cellular ATP content less effectively in normal endothelial cells than free DOX did.

### Disorders of redox homeostasis of human breast cancer cells after treatment with DOX–Tf conjugate

**Figure 3:**
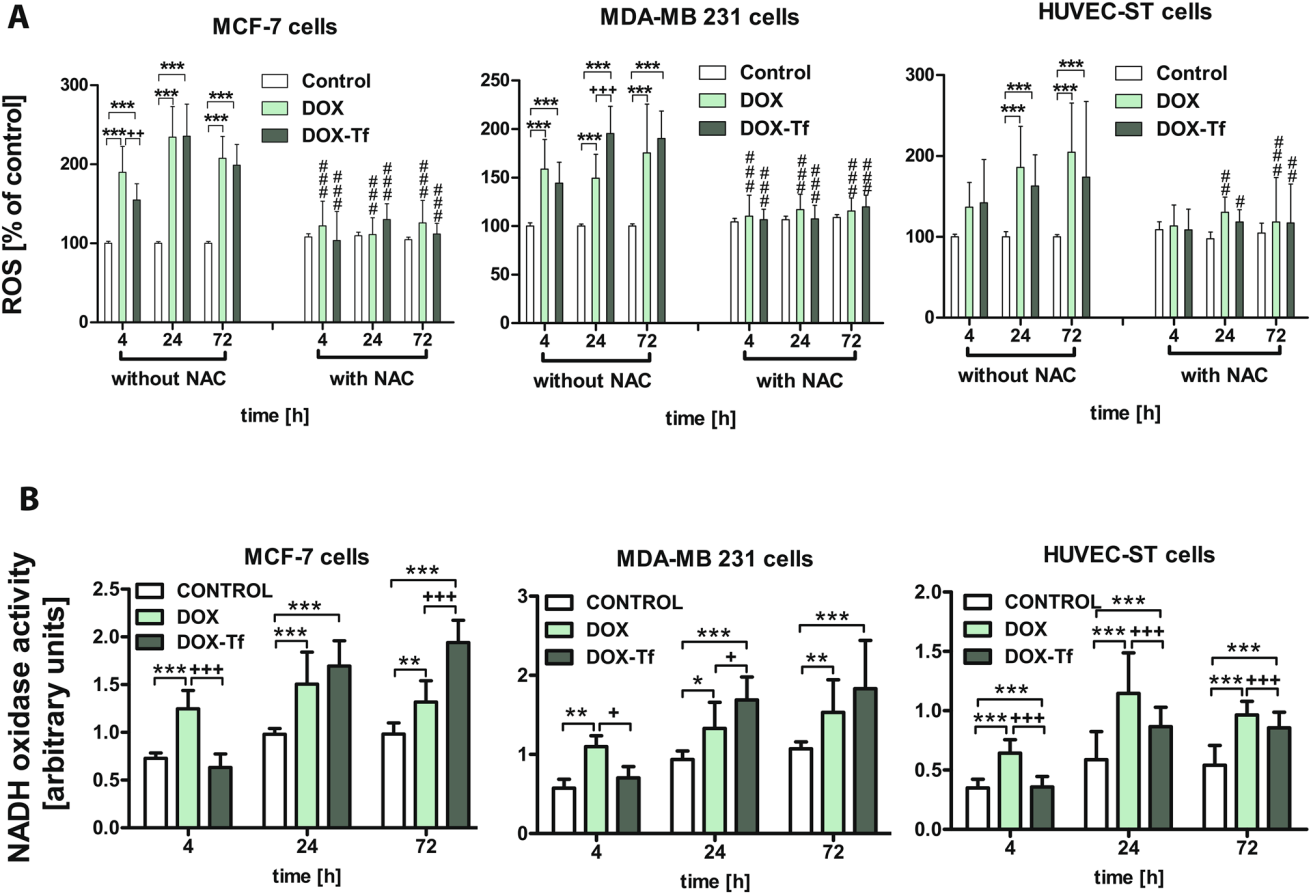
Oxidative stress markers in MCF-7, MDA-MB-231 and HUVEC-ST cell lines treated with DOX–Tf conjugate or DOX alone for 4, 24 and 72 h. (**A**) Cellular ROS level assessed by H_2_DCFDA oxidation after treatment of MCF-7, MDA-MB-231 and HUVEC-ST cells with DOX–Tf conjugate or free DOX (IC_50_ dose) for 4, 24 and 72 h in the absence or presence of ROS scavenger NAC (5 mM). (**B**) Activity of NADH oxidase in MCF-7, MDA-MB-231 and HUVEC-ST cells was measured after 4, 24 and 72 h exposure of cells to free DOX and DOX–Tf conjugate. Data represent mean ± SD of four independent experiments. *p < 0.05, **p < 0.01, ***p < 0.001 denote a statistically significant difference compared with control cells; ^#^p < 0.05, ^##^*p < *0.01, ^###^*p < *0.001 denote a significant difference observed between cells incubated with DOX in comparison to DOX–Tf conjugate; ^**+**^p < 0.05, ^**++**^*p < *0.01, ^**+++**^*p < *0.001; n = 6: difference between expression in the cells treated with DOX or DOX–Tf.

Intrigued by the differential effects of DOX–Tf and free DOX on the viability of human breast cancer lines and their intracellular ATP levels, we aimed to identify the mode of oxidative stress induced by the conjugate. Keeping in mind that the most likely mechanism of DOX antitumor activity is the generation of free radicals, we checked whether the same properties were possessed by DOX–Tf. We measured the accumulation of cellular ROS using H_2_DCFDA and subjecting the cells to 4, 24 and 72 h treatment with DOX or DOX–Tf. ROS levels were significantly increased by both DOX alone as well as DOX–Tf in both breast cancer cell lines, whereas free radical production reached a higher level after incubation of HUVEC-ST cells with free DOX (Fig. 3A). Some differences between these two forms of DOX and the predominant role of DOX–Tf in ROS production were mostly observed in MDA-MB-231 cells after 24 and 72 h incubation. At these times, the DOX–Tf-mediated level of ROS increased by about 90%. Pretreatment of the investigated cells with 3 mM N-acetyl cysteine (NAC) efficiently diminished the drug-induced elevation of ROS levels (Fig. 3A).

To examine further the oxidative stress markers triggered by DOX–Tf conjugate, we assessed NADH oxidase activity, one of the key enzymes involved in production of superoxide free radicals. Alterations in this enzyme activity were measured at 4, 24 and 72 h after incubation of breast cancer cell lines with DOX or DOX–Tf (Fig. 3B). Cell exposure to the IC_50_ concentration of the drugs resulted in time-dependent increase of NADH oxidase activity and reached a maximal level if cells were cultured in the presence of DOX–Tf for up to 72 h (1.94 ± 0.23 and 1.83 ± 0.16 U, respectively). After 24 and 72 h of treatment with both forms of DOX, the increase in NADH oxidase activity was higher with DOX–Tf conjugate than DOX alone. Under the same conditions, NADH oxidase activity was considerably lower in endothelial HUVEC-ST cells (0.85 U) treated with the conjugate.

### DOX–Tf significantly diminishes MMP of estrogen-positive and -negative breast cancer cells

**Figure 4:**
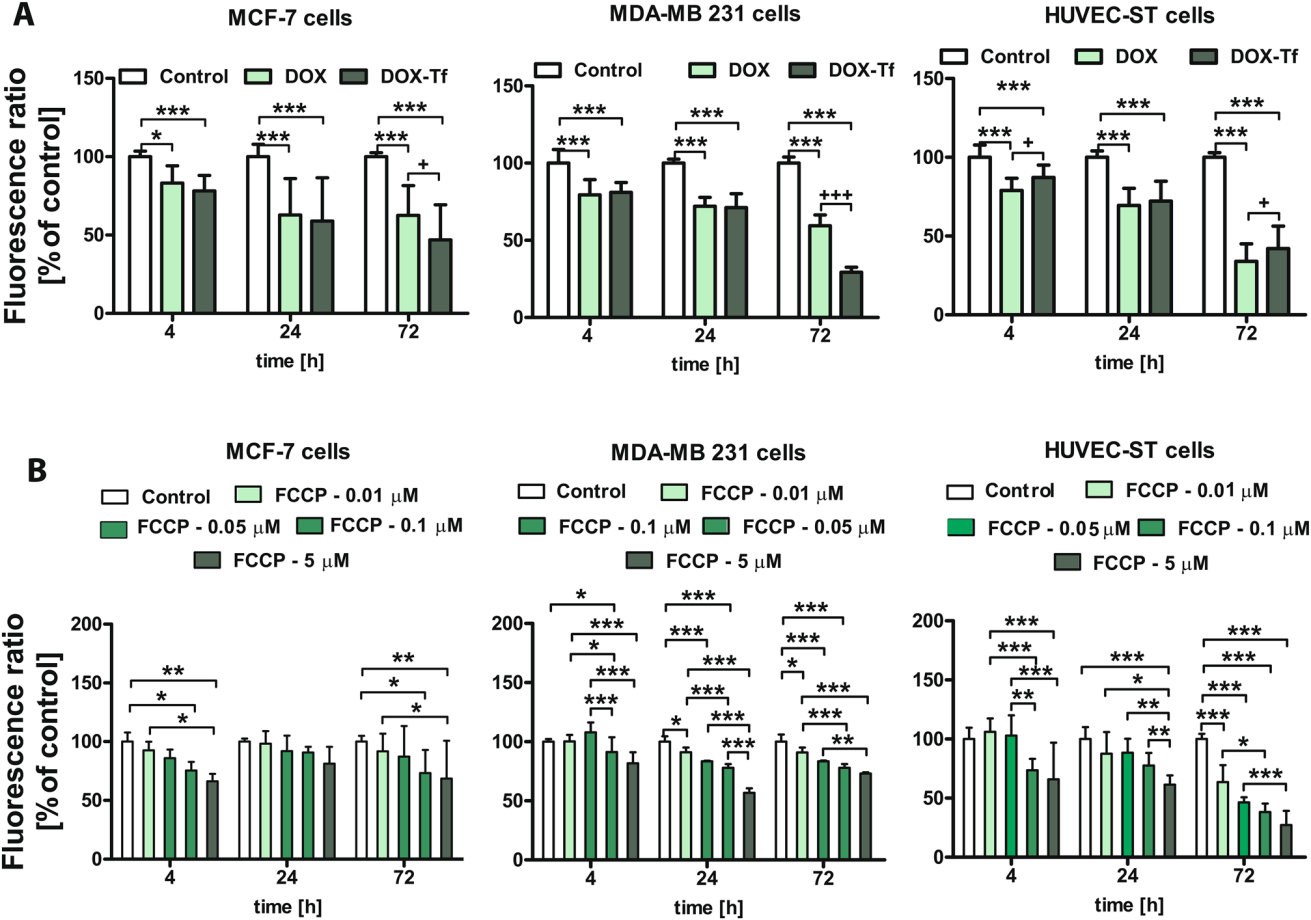
Changes in MMP of MCF-7, MDA-MB-231 and HUVEC-ST cells. (**A**) Effect of DOX–Tf conjugate and free DOX on MMP in human breast cancer cells and endothelial cells. MMP of cells treated with IC_50_ concentrations of DOX–Tf or DOX alone (after 4, 24 and 72 h) was estimated with JC-1. Each result represents mean ± SD of four independent experiments, related to fluorescence of the control sample assumed as 100%. *p < 0.05 significantly different compared to the respective untreated cells; ^+^*p < *0.05, significant differences between samples incubated with DOX or DOX–Tf. (**B**) Collapse of MMP in human breast cancer cells and HUVEC-ST endothelial cell line incubated with FCCP for 4, 24 and 72 h. Fluorescence ratio of JC-1 dimers/JC-1 monomers in control cells was assumed as 100%. Results are presented as mean ± SD of four independent experiments. *p < 0.05, **p < 0.01, ***p < 0.001 denote statistically significant changes in comparison with the control cells (not treated with FCCP) taken as 100%.

**Figure 5:**
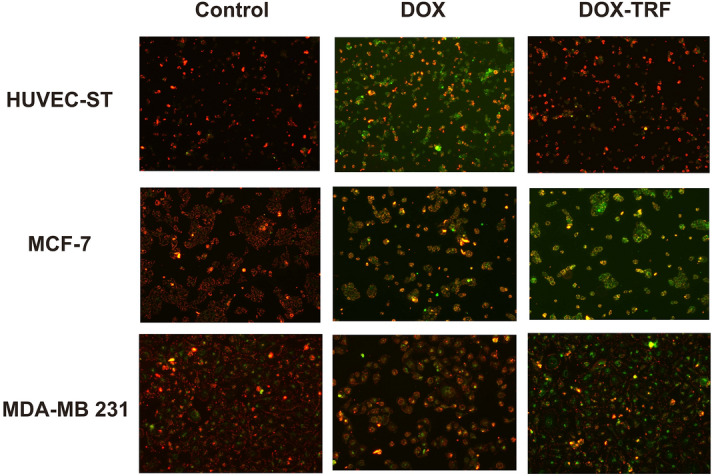
Fluorescent microscopy images of control cells incubated with PBS, and cells treated with IC_50_ concentrations of DOX or DOX–Tf for 24 h. Red fluorescence of JC-1 dimers was present in the cell areas with high MMP, while green fluorescence of JC-1 monomers was prevalent in the areas with diminished MMP. JC-1-stained cells were visualized with an inverted fluorescence microscope (Olympus IX70, Japan); 400 × magnification.

We determined which types of molecular consequences were related to excess production of ROS induced by DOX–Tf. It has been proved that imbalance of redox homeostasis affects mitochondrial function. We therefore measured the alteration of MMP (Δψm) after treatment of breast cancer and noncancer cell lines with DOX or DOX–Tf for 4, 24 and 72 h. As a positive control, a protonophoric uncoupler of oxidative phosphorylation (FCCP) was used prior to labeling the cells with JC-1^21^. As expected, a profound fall in MMP was observed if cells were incubated with FCCP, which reflected a decrease in the JC-1 dimer to JC-1 monomer fluorescence ratio (Fig. 4B). We observed that free DOX or DOX–Tf induced time-dependent changes in Δψm (Fig. 4A). In noncancer HUVEC-ST cells, Δψm was reduced after 4 h incubation with free DOX (79%) and reached maximal collapse when treatment continued for up to 72 h (34%). A decrease in the fluorescence intensity of JC-1 was comparable for cancer cells treated with DOX alone and DOX–Tf conjugate. However, the largest drop in MMP was observed in MDA-MB-231 cells, which after 72 h incubation with DOX–Tf, decreased by nearly 25% compared to the control value. Additionally, Δψm in the DOX–Tf-treated cells was monitored by fluorescence microscopy. DOX–Tf treatment caused a remarkable increase in green fluorescence of JC-1 monomers in breast cancer cells, indicating a reduction in MMP (Fig. 5). In contrast, the red fluorescence of JC-1 dimers was seen mainly in untreated (control) cells with a high MMP.

### DOX–Tf conjugate alters oxygen consumption rates of human breast cancer cells

**Figure 6:**
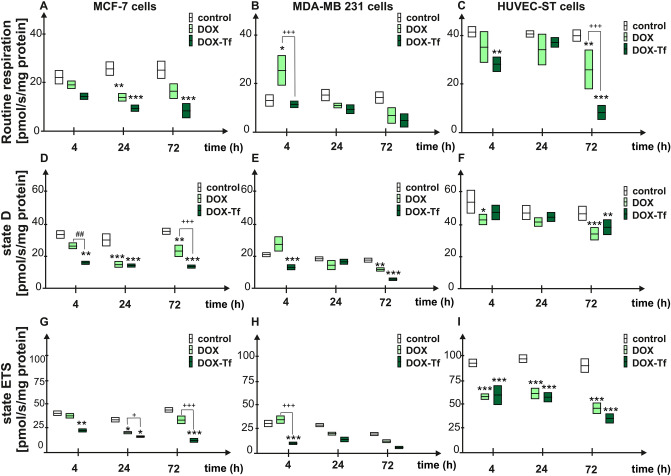
Mitochondrial respiration of MCF-7, MDA-MB 123 and HUVEC-ST cells after incubation with DOX–Tf. Data are expressed as pmol O_2_/s/mg protein in permeabilized cells measured after DOX and DOX–Tf treatment with IC_50_ concentrations. The following mitochondrial states were evaluated: routine respiration (A–C) State D (D–F) and State ETS (G–I) after 4, 24 and 72 h incubation with DOX or DOX–Tf. Data were expressed as mean ± SD, n = 3. *p < 0.05, **p < 0.01, ***p < 0.001 denote statistically significant changes in comparison with the control untreated cells; ^+^*p < *0.05, ^++^*p < *0.01, ^+++^*p < *0.001 significant differences between samples incubated with DOX or DOX–Tf.

Since we observed a collapse of the MMP as well as increased ROS production triggered by DOX–Tf treatment, we determined the exact extent to which mitochondrial functions were influenced by DOX–Tf conjugate two breast cancer cell lines. We monitored different mitochondrial bioenergetics states of MCF-7 and MDA-MB-231 cells as well as HUVEC-ST endothelial cells (Fig. 1A). The cells were incubated with free DOX or DOX–Tf (at IC_50_ concentration) for 4, 24 and 72 h. We observed a significant difference in the mitochondrial routine respiration between cancer and noncancer cells (Fig. 6A–C). MDA-MB-231 cells displayed reduced basic respiration in comparison to MCF-7 or HUVEC-ST cells. Besides, this in vitro model of triple-negative breast adenocarcinoma was the most sensitive to both forms of DOX. The respiration of MDA-MB-231 cells reached higher levels following 4 h incubation with DOX compared to DOX–Tf, which suggests the involvement of mitochondrial stress response pathways immediately after free DOX treatment.

In order to assess the cellular ability to convert ADP to ATP during mitochondrial OXPHOS, we evaluated State D during mitochondrial respiration. The slowest rate of ATP production was observed in MCF-7 cells treated with both forms of DOX (independently of incubation time) (Fig. 6D–F). Additionally, MCF-7 cell line was more resistant to DOX–Tf toxicity than estrogen-negative breast cancer cells.

In contrast to cancer cells, HUVEC-ST cells displayed a reduction in State D only after incubation with free DOX for 4 and 72 h. This observation is attributed to higher basic oxygen respirometric activity of HUVEC-ST cells in comparison to breast cancer cell lines.

Finally, to evaluate the electron transport efficiency of mitochondria in the presence of DOX or DOX–Tf, we assessed the changes in the level of State E. MDA-MB-231 cells were less resistant to both forms of DOX than MCF-7 cells were (Fig. 6G–I). However, if treatment was continued for up to 72 h, the electron transport rate through the inner mitochondrial membrane was significantly inhibited in all cell lines.

### Mitochondrial bioenergetics are changed in a DOX–Tf-dependent manner

**Figure 7:**
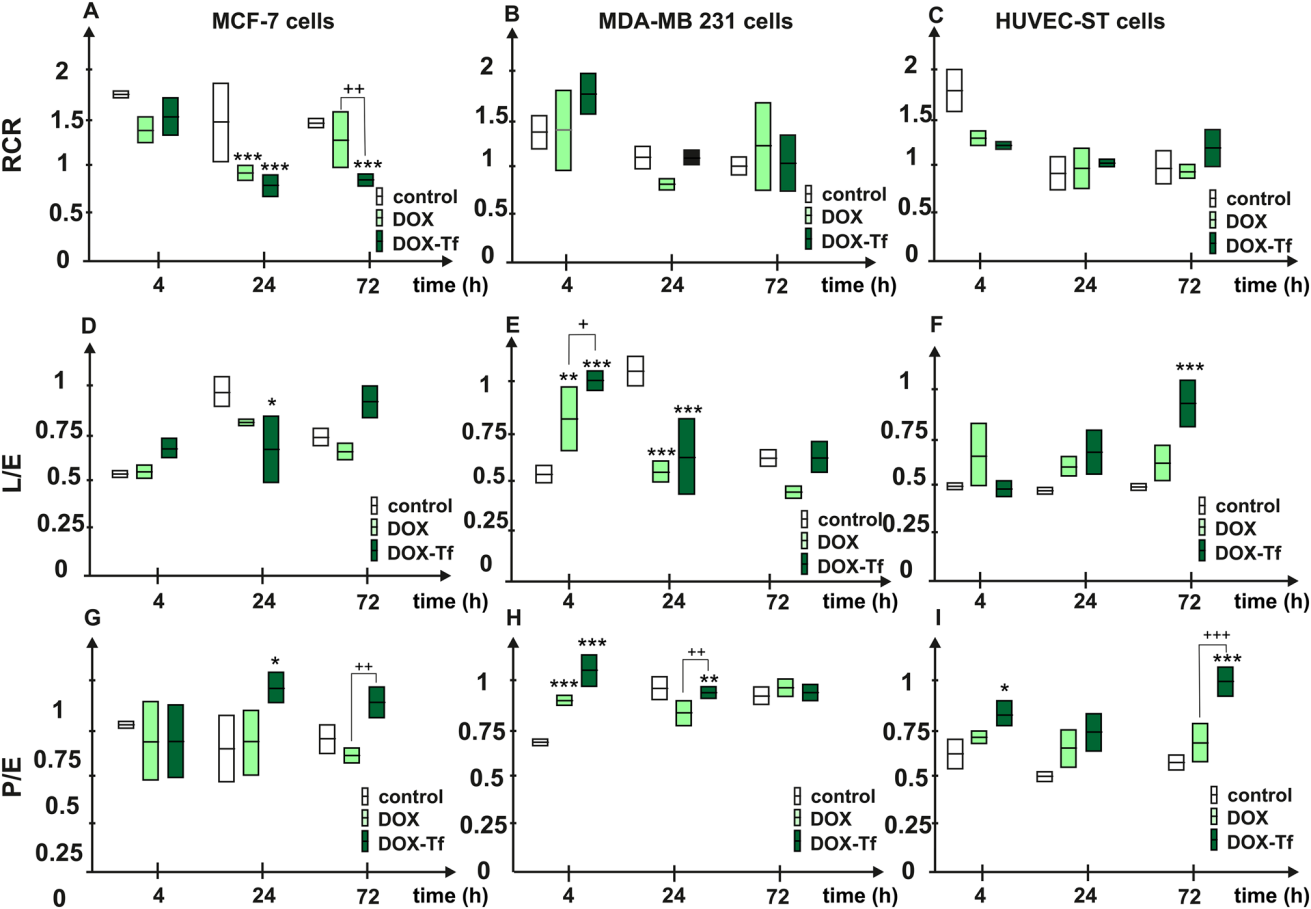
Selected parameters of mitochondrial bioenergetics in human breast cancer cell lines (MCF-7 and MDA-MB-231) and noncancer HUVEC-ST cells. The following mitochondrial parameters were evaluated: RCR (A–C) L/E, (D–F) and P/E (G–I). Measurement was performed after 4, 24 and 72 h incubation with DOX alone or DOX–Tf conjugate (IC_50_ doses). Data are expressed as mean ± SD, n = 3, *p < 0.05, **p < 0.01, ***p < 0.001 denote statistically significant changes in comparison with the control untreated cells; ^+^*p < *0.05, ^++^*p < *0.01, ^+++^*p < *0.001 significant differences between samples incubated with DOX or DOX–Tf.

To assess whether DOX and DOX–Tf altered the integrity of the mitochondrial membrane, the Respiratory Control Ratio (RCR) parameter was measured in the presence of both forms of DOX. The highest reduction of RCR was observed in MCF-7 cells (Fig. 7A–C). Integrity of the mitochondrial membrane was reduced in parallel with prolonged incubation with DOX or DOX–Tf; however, substantial impairment was detected in the DOX–Tf-conjugate-treated cells. A similar drop in RCR was observed in MDA-MB-231 cells incubated with both forms of DOX, but no significant changes were seen in noncancer, endothelial cells.

To assess how strong and reversible mitochondrial stress triggered by DOX or DOX–Tf was, we measured Leak Control Ratio (L/E) parameter, which describes the cellular ability to uncouple the mitochondria. Surprisingly, the adaptive process of cancer cells to endure the toxic effect of DOX or DOX–Tf revealed that MDA-MB-231 cells were more sensitive than MCF-7 cells to DOX. Untreated MCF-7 breast cancer cells had lower L/E parameter after 24 h than cells treated with DOX or DOX–Tf conjugate (Fig. 7D–F). In contrast, the coupling state of noncancer HUVEC-ST cells displayed resistance to DOX treatment, regardless of duration of incubation, although substantial reduction of this parameter was observed after 24 and 72 h treatment with DOX–Tf. Apart from RCR and L/E, the last but not least mitochondrial parameter that provides information about mitochondrial condition after treatment is the Phosphorylation System Control Ratio (P/E) parameter. The lowest OXPHOS capacity was observed in MDA-MB-231 cells compared to MCF-7 and HUVEC-ST cells (Fig. 7G–I). The time-dependent exposure to DOX and DOX–Tf conjugate revealed that the adverse effect of DOX–Tf is strongly time dependent. The resistance of cells to DOX toxicity regarding the mitochondrial stress, was noted for MDA-MB-231 and MCF-7 cancer cells, after 24 and 72 h treatment. The OXPHOS capacity of HUVEC-ST cells was diminished after DOX–Tf conjugate treatment for 4 and 72 h and remained completely unchanged if endothelial cells were incubated with DOX.

### Release of cytochrome c from mitochondria is triggered by DOX–TfF treatment

We proved that DOX–Tf conjugate inhibited the cellular rate of energy production (Fig. 6) and reduced OXPHOS efficiency (Fig. 7). Therefore, the mitochondrial cytochrome c release was examined in breast cancer and endothelial cells after 4, 24 and 72 h incubation with both forms of DOX. The mitochondria of HUVEC-ST cells did not release cytochrome c (Table 1). However, cytochrome c leakage was observed in cancer cells after treatment with DOX or DOX–Tf conjugate, mostly after 24 and 72 h. These results suggest that both forms of DOX increased mitochondrial membrane permeability, provoking damage and triggering disorders in voltage-dependent anion channel function.

**Table 1:** Integrity of the inner mitochondrial membrane that refers to the cytochrome c leakage in MCF-7, MDA-MB-231 and HUVEC-ST cell lines after exposure to IC_50_ concentrations of DOX or DOX–Tf conjugate.

Time (h)		HUVEC- ST	MCF− 7	MDA− MB 231
4	Control	− 4.60 ± 2.60	1.37 ± 6.13	− 3.76 ± 4.04
	DOX	− 1.02 ± 3.70	0.51 ± 6.70*	5.76 ± 10.56
	DOX-TRF	− 1.81 ± 5.94	7.10 ± 10.23	3.60 ± 6.27
24	Control	− 0.49 ± 3.37	2.66 ± 2.43	2.27 ± 2.20
	DOX	− 0.53 ± 4.03	40.04 ± 18.90	11.45 ± 6.13**
	DOX-TRF	0.38 ± 5.95	21.83 ± 12.46	45.38 ± 12.28
72	Control	− 1.38 ± 1.75	0.13 ± 5.42	− 0.01 ± 7.62
	DOX	1.59 ± 4.81	19.63 ± 13.23**	57.91 ± 41.29***
	DOX-TRF	1.75 ± 7.24	41.94 ± 14.13***	17.21 ± 8.09^+^

Data are expressed as the percentage values and shown as mean ± SD, n = 3. *p < 0.05, **p < 0.01, ***p < 0.001 denote a significant difference compared to the respective untreated cells, and ^+^*p < *0.05, ^++^*p < *0.01, ^+++^*p < *0.001 denote significant differences between samples incubated with DOX or DOX–Tf.

### DOX–Tf exerts differential expression of genes involved in mitochondrial stress

**Figure 8:**
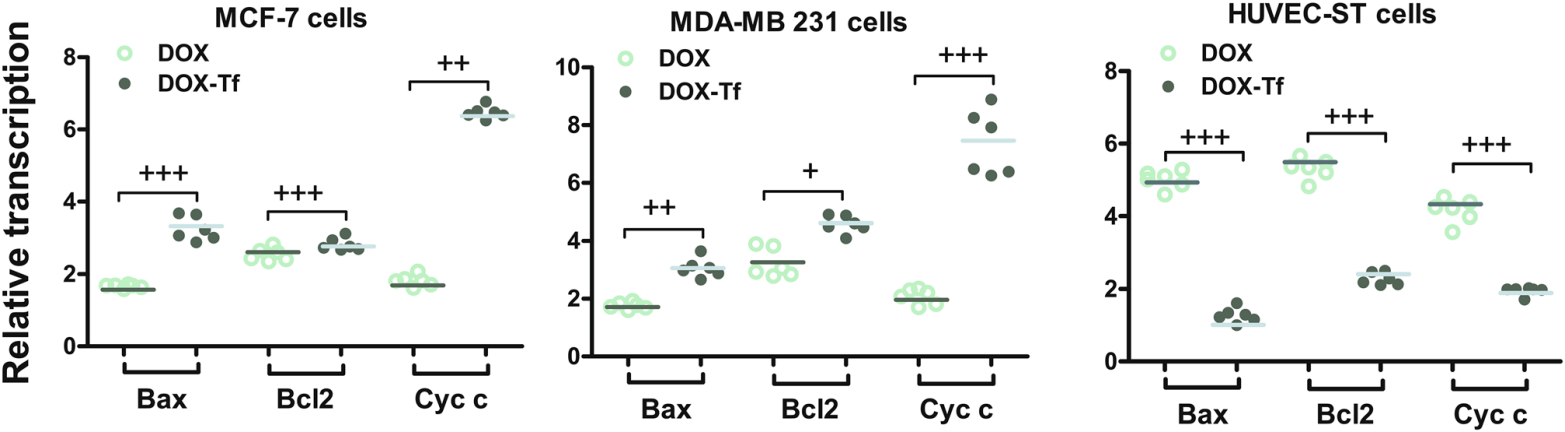
Expression of Bax, Bcl2 and Cytochrome c gene transcripts (relative to HPRT1 housekeeping gene) in MCF-7, MDA-MB-231 and HUVEC-ST cells exposed to IC_50_ concentrations of DOX or DOX–Tf. Asterisks refer to the level of significant (^+^*p* < 0.05, ^++^*p < *0.01, ^+++^p < 0.001; n = 6) difference between expression in the cells treated with DOX or DOX–Tf.

As treatment with DOX–Tf conjugate potently induced oxidative and mitochondrial stress, we investigated whether expression of key mitochondrial proteins was also altered after DOX–Tf conjugate treatment. Transcription of Bax, Bcl-2 and cytochrome c always increased in the DOX–Tf-treated cells (Fig. 8). Breast cancer cells incubated with DOX–Tf showed the maximal values of cytochrome c expression at the mRNA level (6.5 and 7.4 times higher for MCF-7 and MDA-MB-231 cells, respectively) in comparison to control, non-treated cells). In noncancer cells, free DOX increased mRNA level of Bax, Bcl-2 and cytochrome c 5, 5.3 and 4.2 times, respectively, whereas the changes induced by DOX–Tf were not so substantial.

## Discussion

The loss of DOX therapeutic efficacy during conventional breast cancer treatment is strictly related to: (1) development of drug resistance; (2) heterogeneity of the cancer tissue; and (3) high rates of mutations and selection during carcinogenesis^22^. A number of vehicles have been proposed to increase the target effect of DOX^23^, but there is still a lack of totally satisfactory delivery methods. In this study, we proposed DOX bound to Tf, as a new drug delivery system that seems to be a promising tool for enabling accumulation of nonselective chemotherapeutic drugs at target areas, while reducing the exposure of normal healthy cells to these drugs^24^. Our primary goal was to investigate the cytotoxic potential of DOX–Tf conjugate and the mitochondrial stress parameters that this conjugate can induce. We demonstrated that DOX–Tf, used as an effective drug delivery system in human breast cancer cells, significantly affected mitochondrial metabolism. We used two different breast cancer cell lines, MCF-7 and MDA-MB-231, to study mitochondrial stress responses and the cytotoxic effect of DOX–Tf conjugate. In parallel, the experiments were performed on normal HUVEC-ST cell line. It is worth underlining that newly developed drug delivery systems may induce crosslinking of cellular receptors, initiate signaling processes, induce structural alterations at the cell surface, and interfere with normal endothelium function. Moreover, the physicochemical properties of natural and synthetic DOX carriers can cause undesirable toxic effects that require assessment of their interaction with normal, noncancer cells^25^. In our study, MTT assay revealed that MDA-MB-231 was the most sensitive cell line and MCF-7 the least sensitive after 72 h incubation with free DOX as well as DOX–Tf conjugate. In contrast to the tested cancer cell lines, which were significantly affected by DOX–Tf treatment, a lesser decrease in the rate of formazan crystal formation was observed in HUVEC-ST cells. These cells were not completely resistant to DOX–Tf treatment, but showed a delayed response, as confirmed by the observation of ATP measurements.

In agreement with the idea that DOX increases ROS production^26^ and activates many signaling pathways, we estimated the oxidative stress markers that DOX–Tf attenuated. Our data confirmed that DOX–Tf conjugate mediated free radical generation more effectively than free DOX. Besides the effect on ROS induction, DOX–Tf conjugate treatment increased NADH oxidase activity in a time-dependent manner. This finding is in agreement with our previous study on leukemia cells that showed the maximal increase of NADH oxidase activity after 24 and 48 h incubation with conjugate in CCRF-CEM and K562 cells^27^.

It should be noted that, even though DOX–Tf treatment directly led to upregulation of cellular ROS production, we did not observe a great difference in the oxidative stress markers between the two investigated breast cancer cell lines. Our previous data clearly confirmed that neither Tf structure, molecular weight and molecular functions nor DOX properties are changed during conjugation^15^. However, it is still not clear why there is such a strong difference in the sensitivity of the cells to the DOX–Tf conjugate. Firstly, both MCF-7 cells as well as MDA-MB-231 cells have high demand for iron ions and overexpress TfR differently. Corte-Rodríguez et al. observed a fourfold increase in the CD71 protein expression of triple negative breast cancer cultures in comparison to MCF-7 cells^19^.

Additional explanation of these discrepancies in breast cancer cells’ sensitivity may be supplied by Theodossiou et al.^28^, who reported a high level of glutathione peroxidase 4 in MCF-7 cell line. They also found that there is more efficient glutathione-mediated antioxidant defense in MCF-7 than in MDA-MB-231 cells. The strong antioxidant defense system of MCF-7 cells (ER positive) may be connected with estrogen-dependent pathways, which makes these cells highly resistant to conventional chemotherapy^29^. Our data point to the alterations in cellular redox capacity as an important mechanism of DOX–Tf cytotoxicity. Because OXPHOS is a major source of free radical production after DOX treatment, we estimated whether mitochondrial membrane depolarization was connected to the DOX–Tf-triggered disturbances of cellular redox homeostasis. Indeed, when the cells were labeled with JC-1, after treatment with free DOX or DOX–Tf conjugate, we noticed a dramatic decrease in MMP in a time-dependent manner. In vitro models of non-small cell lung cancer (A549 cells) as well as hepatocellular carcinoma (HepG2 cells) have shown the same predominant role of DOX–Tf conjugate^16^. These observations are in agreement with the previously discussed changes in the activity of mitochondrial oxidoreductase–NADH oxidase. This enzyme generates superoxide by transferring electrons from NADPH inside the cell across the membrane and coupling these to molecular oxygen to produce superoxide anions^30^. Consequently, the augmented ROS production that was indirectly intensified by action of NADH oxidase provoked an assessment of mitochondrial metabolism in breast cancer cells exposed to free DOX as well as Tf-bound DOX. Earlier studies have shown that DOX as a constituent of various nano-delivery systems exerts more toxicity towards mitochondria than its non-triggered counterpart^13,31^. To the best of our knowledge, no research has been reported so far concerning the influence of DOX–Tf conjugate on mitochondrial bioenergetics. It was proved in our previous study that Tf bound DOX was initially often located in the cytosol of the human leukemia cells, possibly in endosomal—like related structures. The microscopic observation of normal lymphocytes during drug treatment also showed a different location of DOX and DOX–Tf, proving that the mechanism of plasma membrane passage and subsequent intracellular routing are different between both drugs. A predominantly cytoplasmic location of DOX–Tf potentially exposes the conjugate to bioreductive processes that are known to play an important role in DOX cytotoxicity^15^. There are many strategies used to bring compounds into mitochondria that disturbs its function. For instance, DOX has been reported to cause mitochondrial dysfunction, energy stress via disruption of the electron transport chain (ETC)^32^. Keeping in mind Neuzil’s classification of anticancer agents that act via mitochondrial destabilization^33^, we checked whether Tf bound DOX can distract the homeostasis of mitochondria indirectly. . First, our results confirmed that there is a large difference in rate of mitochondrial OXPHOS in the two breast cancer cell lines that might be related to their different basal redox homeostasis and antioxidant defenses. Second, the bioenergetics profile of MCF-7 as well as MDA-MB-231 cells provided further evidence that cancer should be considered not only as a disorder of uncontrolled proliferation but also as a metabolic disease, although Warburg postulated that tumor cells have defective mitochondrial OXPHOS and therefore rely more on aerobic glycolysis^9^.

We demonstrated that DOX–Tf as well as free DOX triggered mitochondrial dysfunction in breast cancer cells in a time-dependent manner. Surprisingly, a reversible effect of DOX after 24 h continuous incubation was seen in both breast cancer cell lines. This finding indicates that only longer time of exposure to DOX may achieve a desirable cytotoxic effect. Our observation is in agreement with that of Gilliam et al.^34^, who revealed that rodent skeletal muscle shows the characteristic markers of mitochondrial and oxidative stress after 72 h continuous incubation with DOX, while the recovery of mitochondria states is evident at 24 h. The explanation of these data may be associated with the overexpression of mitochondrial heme oxygenase-1 that is involved in prevention of mtDNA damage and inhibition of mitochondria-dependent programmed cell death pathways^35^.

Our previous finding, that DOX–Tf conjugate is able to prolong the cytotoxic properties of DOX and extend the presence of drug inside cancer cells^15^, is strictly related to the observation of mitochondrial membrane damage. In the present study, we found cytochrome c leakage from mitochondria only in tumor cells after 24 and 72 h treatment with DOX or DOX–Tf but the integrity of the mitochondrial membrane in normal cells remained intact. We therefore assume that transformation of the mitochondrial bioenergetics system in cancer cells towards OXOPHOS reduction makes them more sensitive to DOX–Tf conjugate cytotoxicity.

We found that DOX–Tf conjugate was > 17-fold more toxic to MDA-MB-231 cells than to MCF-7 cells. The production of ROS and redox imbalance following addition of DOX–Tf conjugate confirmed that oxidative stress induction is one of the cytotoxic consequences of treatment. Free radicals produced by DOX–Tf mediated the alterations of mitochondria bioenergetics and caused mitochondrial damage. DOX–Tf conjugate is a promising drug delivery system that strongly affects mitochondrial homeostasis. This finding refers only to breast adenocarcinoma cells since noncancer HUVEC- ST cells are more resistant to DOX–Tf conjugate treatment.

In conclusion, we demonstrated that DOX–Tf showed a different degree of toxicity for two breast cancer cell lines MCF-7 and MDA-MB-231 and noncancer HUVEC-ST endothelial cells. DOX–Tf conjugate affected the fate of the cancer cells and noncancer endothelial differently. We revealed that MDA-MB-231 cell line was the most sensitive and noncancer HUVEC-ST cells were the least sensitive of these three investigated cell lines. DOX–Tf induced ROS and triggered a decrease in MMP. This is in contrast to what we noticed with HUVEC-ST cells, where we detected a minor effect of DOX–Tf. Furthermore, we detected that DOX–Tf altered the mitochondrial bioenergetics and impaired the mitochondrial membrane integrity. In summary, we observed that DOX–Tf conjugate helped to increase intracellular concentration of the drug and consequently enhanced its anticancer activity. These results demonstrate that the mechanism of action of DOX–Tf is cell-type dependent, and that its cytotoxicity should be tested specifically on the cancer types one wants to treat.

## Methods

### Materials

Malate, glutamate, succinate, rotenone, oligomycin, antimycin A, digitonin, ADP, carbonyl cyanide p-trifluoromethoxyphenylhydrazone (FCCP), 3-(4,5-dimethylthiazol-2-yl)-2,5-diphenyltetrazolium bromide (MTT), 5,5′,6,6′-tetrachloro-1,1′,3,3′-tetraethyl-benzimidazolcarbocyanine iodide (JC-1) and all reagents for carrying out the conjugation procedure we obtained from Sigma (Darmstadt, Germany). DOX was obtained from Sequoia Research Products (Pangbourne, UK) and free drug was coupled to Tf using the modified conjugation procedure developed by Berczi et al.^36^ (Patent claim no. WIPO ST 10/C PL 402,896). Subsequently, an obtained conjugate was analyzed by mass spectrometry^15^. Dulbecco’s modified Eagle’s medium (DMEM), fetal bovine serum (FBS), penicillin streptomycin, L-glutamine, and phosphate-buffered saline were from Lonza (Lievres, Belgium), whereas all tissue culture dishes were purchased from PAA (Berlin, Germany). 2′7′-Dichlorodihydrofluorescein diacetate (H_2_DCF-DA) and JC-1 were obtained from Life Technologies (Warrington, UK). All the other reagents and solvents were of the highest analytical reagent grade.

### Cancer cell lines

Two different cell lines of human breast adenocarcinoma: MDA-MB-231 (triple-negative breast cancer cells, without ER) and MCF-7 (with ER). The cell cultures were obtained from the Global Bioresource Center ATCC. The cells were cultured in DMEM with extra L-glutamine (4 mM), penicillin (100 U/mL), streptomycin (100 μg/mL), and 10% v/v FBS in standard conditions: 37 °C, 100% humidity, 5% CO_2_ and 95% air as we described before^16^. In parallel, the experiments were carried out on normal HUVEC-ST cell line (human umbilical vein endothelial cells immortalized by transfection with both SV40 large/small T antigens and the catalytic subunit of human telomerase). The cells were a kind gift from Department of Molecular Biophysics, University of Lodz, Poland. HUVEC-ST cells were cultured in OptiMEM supplemented with 3.5% FBS, 100 U/mL penicillin and 100 μg/mL streptomycin (37 °C, 5% CO_2_).

### Cytotoxicity assay

Cells were seeded on 96-well plates at a density of 2500 (HUVEC-ST) or 10 000 (MDA-MB-231 and MCF-7) per well. After 24 h, free DOX or DOX–Tf conjugate was added to the growth medium in graded concentrations and then incubated at 37 °C for 72 h. The MTT assay was performed five times to measure the cytotoxic effects of the above agents. The dark-colored crystals produced by viable cells were solubilized with dimethylsulfoxide. The absorbance was measured at 580 nm (analytical wavelength) as well as 720 nm (reference wavelength) and the IC_50_ values (drug concentration required for 50% growth inhibition) were determined in the two different cell lines, using GraphPad Prism 4.03 software (GraphPad Inc.).

### Determination of bioluminescence of ATP concentration

The luciferin–luciferase system was used for intracellular ATP measurement^37^. The cellular ATP was extracted by adding 100 µL boiling water to the MCF-7, MDA-MB 231 and HUVEC-ST cell monolayers seeded on 96-well plates and treated with DOX or DOX–Tf at IC_50_ concentrations, calculated on the basis of MTT assay. After vortexing and centrifugation (12,000 g for 5 min at 4 °C), 20 µL of the received cellular extract was used for bioluminescence measurement, whereas 10 µL was taken to measure protein concentration. In brief, 20 µL of the measured ATP solution was mixed with 100 µL of LAB buffer (250 mM glycylglycine, 2 mM EGTA, 2 mM MgCl_2_, 0.04% bovine serum albumin, 7.5 mM dithiothreitol, 0.015 mg/mL luciferin and 0.01 mg/mL luciferase, pH 7.7 reconstituted with distilled water) in each well of the microplate. The ATP bioluminescence was measured immediately using a microplate luminometer (Fluoreskan Ascent FL; Labsystems, Farsta, Sweden). The integration time of the luminometer was set at 1 s with normal gain. All reagents were placed on ice before the experiments. The amount of ATP was estimated from a standard curve and results were expressed as mmol ATP/ mg protein.

### ROS estimation

The level of ROS in MCF-7, MDA-MB 231 and HUVEC-ST cells seeded on 96-well plates was measured by monitoring changes in the fluorescence of derivatives of 5 µM H_2_DCF-DA after 4, 24 and 72 h incubation of cells with DOX–Tf or DOX. Assays were performed in modified Hank’s buffered salt solution (HBSS), containing 140 mM NaCl, 5 mM KCl, 0.8 mM MgCl_2_, 1.8 mM CaCl_2_, 1 mM Na_2_HPO_4_, 10 mM HEPES and 1% glucose, pH 7.0. Fluorescence intensity was monitored with a Fluoroskan Ascent FL microplate reader. As treatment with free DOX and DOX–Tf conjugate affected the cell growth, to avoid errors in estimation caused by probable cell detachment, analysis of DNA content was carried out^38^. After measurement, the probe was removed by gentle aspiration, the cell monolayers were washed three times with PBS, and the plates was frozen at − 70 °C. Thawing at room temperature was followed by the addition of 100 µL deionized water per well and a second freezing at − 70 °C. After subsequent thawing, RNA was digested with RNAse and 100 µL of 5 µM propidium iodide was added, the plate was immediately shaken, incubated at room temperature in the dark for 15 min, and the fluorescence was read at 350/620 nm with a Fluoroskan Ascent FL microplate reader.

### NADH oxidase assay

The evaluation of NADH oxidase activity was carried out by measurement of nitroblue tetrazolium (NBT) reduction^27^. MCF-7, MDA-MB 231 and HUVEC-ST cells were treated with DOX–Tf or DOX for 4, 24 and 72 h at IC_50_ concentrations and then washed twice with PBS. Then, 50 µL ice-cold lysis buffer (10 mM potassium phosphate buffer, pH 7.4, 0.1 mM EDTA, 0.1% Triton X-100) was added to the cell suspension and 100 µL NADH solution was added to half of the plate, and to the other half, we added the same amount of NADH oxidase assay buffer (10 mM potassium phosphate buffer, pH 7.4, 0.1 mM EDTA). Finally, 50 µL NBT solution was added and the plate was incubated at 37 °C while mixing for 30 min. Quantitative NBT reduction was measured on a plate reader (Awareness Technology Inc., Palm City, FL, USA) at 560 nm in point mode.

### Measurement of mitochondrial membrane potential (MMP)

The alteration in MMP was assessed by measuring JC-1 fluorescence intensity ratio, as described before^21^. MCF-7, MDA-MB 231 and HUVEC-ST cells were seeded on 96-well black microplates. After 24 h, IC_50_ concentrations of the drugs or 0.01–5 μM FCCP were added to the wells. The cells were incubated with DOX, DOX–Tf or FCCP for 4, 24 and 72 h. At the end of treatment, the medium was removed and the cells were incubated in total darkness with HBSS containing 5 µmol/L JC-1 for 30 min at 37 °C. The fluorescence of both JC-1 monomers and dimers was measured on a Fluoroskan Ascent FL microplate reader using filter pairs of 530 nm/590 nm (dimers) and 485 nm/538 nm (monomers). In parallel, the ratio of JC-1 dimer to monomer in relation to the untreated control cells was monitored by Olympus IX70 fluorescence microscope (Olympus, Tokyo, Japan).

### Measurement of mitochondrial bioenergetics

The parameters of mitochondrial bioenergetics (Fig. 1A) were determined using an Oroboros-2 k oxygraph (Oroboros Instruments, Innsbruck, Austria). Datalab software (Oroboros Instruments) was used for data acquisition and analysis. All measurements were performed at 37 °C in MIRO5 medium (110 mM sucrose, 60 mM K-lactobionate, 0.5 mM EGTA, 1 g/liter BSA essentially fatty acid free, 3 mM MgCl_2_, 20 mM taurine, 10 mM KH_2_PO_4_, and 20 mM HEPES, adjusted to pH 7.1) with continuous stirring at 750 rpm in the presence of mitochondrial substrates and inhibitors, according to the slightly modified procedures that were described previously^39,40^.

MCF-7, MDA-MB-231 and HUVEC-ST cells were seeded on Petri dishes (3,000,000 cells/dish) and cultured for 1 day at 37 °C before treatment with two forms of DOX. DOX–Tf as well as free DOX (IC_50_ concentration) were incubated with the cells for 4, 24 and 72 h at 37 °C. The cells were thoroughly washed with PBS and harvested by trypsin–EDTA (Sigma–Aldrich, St Louis, MO, USA), pelleted, and suspended in respiration buffer (MIR05). The cell suspensions were added to each chamber (Fig. 1B,C) and stabilized under the routine respiration conditions. Digitonin (10 µg/mL) was added to access the mitochondria with different respiratory substrates (the optimal concentration of digitonin was evaluated in an independent set of experiments) (Fig. 1C). Respiration through Complex I was measured by adding glutamate (5 mM) and malate (5 mM), then the maximal respiratory capacity with convergent electron flow through both Complex I and Complex II was achieved by adding succinate (10 mM). Subsequently, ADP addition at saturating concentration (0.25 mM) caused the maximal OXPHOS capacity (or State 3), after which ATP synthase was inhibited by oligomycin (2 µg/mL) to evaluate State 4. In all experiments, the lack of significant increase in respiration after addition of cytochrome c confirmed the integrity of the outer mitochondrial membrane. The maximal electron flow through respiration without OXPHOS (ETS state) was assessed by the stepwise (5–20 µL) titration of FCCP (2.5–10 µM). Uncoupled Complex-I-linked respiration was achieved by adding rotenone (0.5 µM). Finally, the ETS was inhibited by antimycin A (2.5 µM) to obtain the residual oxygen flux (ROX). ROX after antimycin addition was subtracted from the steady-state respiration values. Finally, the respiration values were calculated as the decrease in the oxygen concentration at time measured in closed chambers, and expressed per milligram of protein. The protein concentration in each sample was determined by the bicinchoninic acid assay.

### Quantitative real-time polymerase chain reaction (PCR)

Total RNA from cells treated with DOX–Tf conjugate or DOX, at IC_50_ doses for up to 24 h, was extracted using TRIzol Reagent (Sigma). The concentration and purity of the RNA were determined using a UV–Vis Spectrophotometer (Biotek Eon, Waltham, MA, USA). cDNA was generated using the High-capacity cDNA Reverse Transcription kit (Applied Biosystems, Waltham, Massachusetts, USA) as was described before^16^ and quantitative real-time PCR was performed with the SYBR-green PCR master mix (EURx, Gdansk, Poland) in an Eco Real-Time PCR System (Illumina, San Diego, California, USA). The PCR parameters were as follows: one cycle of 95 °C for 30 s followed by 40 cycles of 95 °C for 3 s and 60 °C for 30 s. Hypoxanthine–guanine phosphoribosyltransferase (HPRT) was used as the endogenous reference gene and the primer sequences used were as follows: B-cell lymphoma 2 (Bcl-2) forward, 5′-GCACGCTGGGAGAAAGGGTACGAT-3′, and reverse, 5′-CACATCTCCAGCATCCCACTCGTA-3′; Bcl-2-like protein 4 (Bax) forward, 5′-CGAAATTCAAAGGATGGGCTCCTGGTT-3′, and reverse, 5′-CGGTTAACCCGGGTAAGAAATGTGCAT-3′; cytochrome c (cyt c) forward, 5′-AGGCCCCTGGATACTCTTACACAG-3′, and reverse, 5′-TCAGTGTATCCTCTCCCCAGATG-3′; HPRT forward, 5′-TGACACTGGCAAAACAATGCA-3′, and reverse, 5′-GGTCCTTTTCACCAGCAAGCT-3′. Target genes were normalized to the reference genes. Relative gene expression levels using the test and reference genes were calculated by the comparative Cq method.

### Statistical analysis

Statistics were calculated using STATISTICA.PL software v.12.5 (StatSoft, Krakow, Poland)^27^. All of the measurements were performed at least in duplicate (3–6 times). The sample size was estimated for type I and type II statistical errors of 0.05 and 0.8, respectively. The data were expressed as mean ± SD. The data were tested for normal distribution with the Shapiro–Wilk test, and variance of homogeneity was verified with the Brown–Forsythe test. Following this testing, the data with a normal distribution were analyzed with parametric tests. The statistical significance between homogeneous groups was estimated using multivariate ANOVA with the planned measurements or post-hoc Tukey test. The post-hoc power of the used tests was checked for each parametric analysis. A statistical power below 80% was considered an invalid outcome, and constructive conclusions were not formulated. Additionally, the viability curves were prepared using the GraphPad Prism 5.0 software. A *p* value of 0.05 was considered significant.

### Consent for publication

The manuscript has been read and approved by all the authors, the requirements for authorship have been met, and each author believes the manuscript represents honest work.

## Data Availability

All data generated or analysed during this study are included in this published article and its additional file.
